# Grafting versus Crosslinking of Silk Fibroin-g-PNIPAM via Tyrosine-NIPAM Bridges

**DOI:** 10.3390/molecules24224096

**Published:** 2019-11-13

**Authors:** Ionut-Cristian Radu, Iuliana-Elena Biru, Celina-Maria Damian, Andreea-Cristina Ion, Horia Iovu, Eugenia Tanasa, Catalin Zaharia, Bianca Galateanu

**Affiliations:** 1 Advanced Polymer Materials Group, Department of Bioresources and Polymer Science, Politehnica University of Bucharest, 011061 Bucharest, Romania; radu.ionucristian@gmail.com (I.-C.R.); celina.damian@yahoo.com (C.-M.D.); I.andreeacristina@yahoo.com (A.-C.I.); horia.iovu@upb.ro (H.I.); 2 Faculty of Applied Chemistry and Materials Science, Politehnica University of Bucharest, 011061 Bucharest, Romania; eugenia.vasile27@gmail.com; 3 Department of Biochemistry and Molecular Biology, Faculty of Biology, University of Bucharest, 050095 Bucharest, Romania; bianca.galateanu@bio.unibuc.ro

**Keywords:** silk fibroin, grafting, crosslinking, chemical modification, poly(*N*-isopropylacrylamide)

## Abstract

This paper reports the synthesis and complex characterization of novel polymeric networks based on the crosslinking of *Bombyx mori* silk fibroin via poly(*N*-isopropylacrylamide) bridges generated by an ammonium cerium nitrate redox system. The research study gives an understanding of the polymerization mechanism in terms of the generation of radical sites, radical growth and termination reaction, as well as the involvement of modifications on silk fibroin structure and properties. The physico-chemical characterization was carried out by FTIR-ATR, X-ray photoelectron spectroscopy and RAMAN spectroscopy with unravelling the chemical modification. The structural characterization and spatial arrangement by secondary structure were carried out by X-ray diffraction and circular dichroism. The thermal behavior and thermal stability were evaluated by differential scanning calorimetry and thermogravimetric analysis. The novel complex polymer network is intended to be used in the field of smart drug delivery systems.

## 1. Introduction

Grafting reactions carried out in aqueous media by radical mechanisms represent a very useful tool for modifying natural polymers due to the possibility of achieving desired properties. This promising technique for the chemical modification of biopolymers through functionalization has gained a lot of attention in recent years due to the ability to tune these polymers by precise approaches for a target application domain [1,2,3,4,5]. The chemical modification of polymers with vinyl monomers has been performed by different mechanisms [6,7,8,9]. One of the most helpful tools for achieving this modification has been ammonium cerium nitrate (ACN), which is used as a redox initiating agent. ACN has proven to be a very efficient radical generator for the graft polymerization of vinyl monomers [10,11,12] into various substrate polymers such as synthetic polymers [12], proteins [13,14,15] and polysaccharides [16,17,18]. This is made possible as a result of good water solubility and the possibility of generating active radicals at desired sites [17]. The reaction mechanism is based on single electron transfer from cerium via the direct generation of radical sites onto polymer backbone. This chemical modification technique can also be used for targeted biomedical applications of natural polymers such as proteins or polysaccharides.

Silk fibroin is a natural polymer with special physical and mechanical properties and a wide range of biomedical applications [19]. The excellent properties of silk fibroin protein are due to biocompatibility, partial biodegradability, low toxicity, processing flexibility and high mechanical strength [20,21,22]. One of the main biomedical applications of silk fibroin is related to drug delivery. The development of silk fibroin nanocarriers has expanded widely over past the decade due to highly controllable properties, composition, structure and architecture. Furthermore, the loading of active agents takes place in aqueous medium, which offers a major advantage in respect to other systems. Thus, silk fibroin has the capacity to develop various nanocarrier systems through its native structure or via different chemical modifications [23,24].

Polymers such as poly(*N*-isopropylacrylamide) (PNIPAM) are known for their thermosensitive response and PNIPAM is considered to be one of the smartest polymers that is sensitive to various environmental factors such as temperature or pH [25,26,27]. In this work, PNIPAM is used as a grafting agent of silk fibroin by generating some inter- and intra-molecular bridges. The mechanism of polymer grafting onto silk fibroin with ammonium cerium nitrate is shown and discussed in detail. This work is the first step in the design of novel smart drug delivery systems, such as self-assembled silk fibroin/PNIPAM nanocarriers, that possess a more controlled release profile compared to other systems based on native silk fibroin. Self-assembled drug delivery systems could be further developed thanks to the good interaction between PNIPAM bridges and hydrophilic active agents such as 5-fluorouracil.

## 2. Results and Discussion

### 2.1. Physico-Chemical Characterization

**Figure 1:**
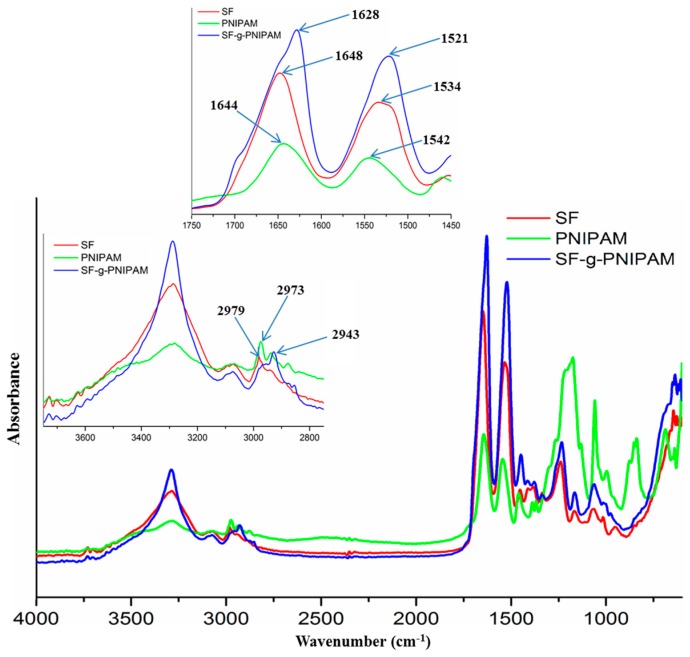
FTIR spectra of silk fibroin (SF), poly(*N*-isopropylacrylamide) (PNIPAM) and SF-g-PNIPAM**.**

FTIR analysis was carried out in order to reveal the modifications generated by the grafting of poly(*N*-isopropylacrylamide) (PNIPAM) onto the silk fibroin chain. The spectra of silk fibroin (SF), PNIPAM and SF-g-PNIPAM (ratio 0.1 *w*/*w* to SF) are shown in Figure 1. The chemical structure of silk fibroin is revealed by the main peak at 3286 cm^−1^, which is specific to the N-H (amide A) and O-H stretching bonds. The next peak at 3076 cm^−1^ is associated with amide B, which is a part of the double Fermi resonance with amide A. The peak at 2979 cm^−1^ is characteristic of C-H asymmetric stretching from methyl groups. The peak at 2936 cm^−1^ is characteristic of C-H asymmetric stretching from methylene backbone. The next FTIR region is very important for the amide I and amide II bands. The amide I assigned to C=O stretching from the backbone amide bonds appears at 1648 cm^−1^. The amide II assigned to N-H bending and C-N stretching appears at 1534 cm^−1^. Peaks at 1447, 1408 and 1335 cm^−1^ correspond to the C-H bending motion in the main backbone and side chains. The amide III at 1232 cm^−1^ is assigned to C-N stretching and N-H bending [28,29,30]. The FTIR spectrum of PNIPAM shows significant peaks at 3289 and 3076 cm^−1^, which are assigned to N-H stretching and N-H bending resonance overtone with amide II. The next peaks are characteristic of C-H asymmetric stretching from methyl at 2973 cm^−1^, and C-H asymmetric stretching from methylene at 2937 cm^−1^. The peak at 1644 cm^−1^ is assigned to amide I from side chain bonds, and the peak at 1542 cm^−1^ is assigned to amide II. The peaks at 1460, 1387 and 1367 cm^−1^ are also assigned to the C-H bending motion, which is related to methyl and isopropyl groups. The last important peak at 1263 cm^−1^ is assigned to amide III [16,25,26]. The FTIR spectrum of SF-g-PNIPAM is similar to the spectrum of crude silk fibroin. The peaks in the area of the C-H stretching motion show an increasing intensity at the 2943 cm^−1^ peaks, which is assigned to C-H asymmetric stretching from methylene groups. The peak’s increasing intensity is explained by the contribution of the grafted PNIPAM, which has a methylene group in the main chain. Major modifications in the spectrum refer to the shifting of the amide I band at 1628 cm^−1^ and amide II band at 1521 cm^−1^. Furthermore, the amide I band changes its shape by the contribution of a new C=O stretching bond revealed by the amide side chains of the grafted PNIPAM. The shifting of the amide I and amide II bands to lower values suggests a serious contribution of the random coil structure and lateral tyrosine to the FTIR spectrum [17]. These aspects show a structural modification in the disordered random coil region by exposing tyrosine amino acid.

**Figure 2:**
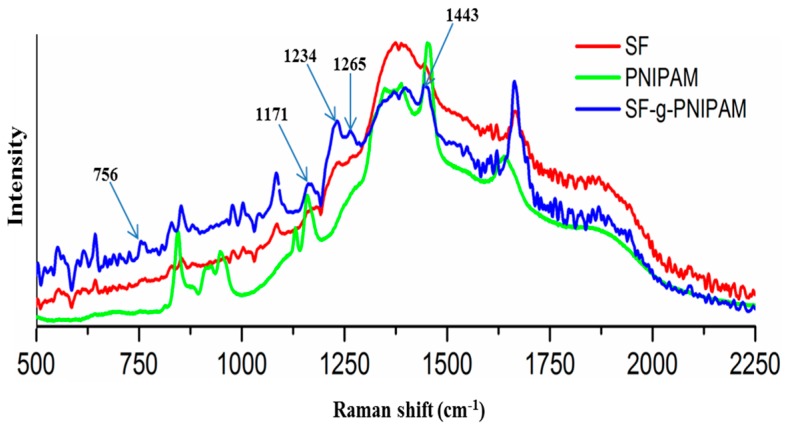
RAMAN spectra of silk fibroin (SF), PNIPAM and SF-g-PNIPAM.

RAMAN spectra of silk fibroin (SF), poly(*N*-isopropylacrylamide) (PNIPAM) and grafted SF-g-PNIPAM (ratio 0.1 *w*/*w* to SF) were collected in the spectral range 500–2250 cm^−1^ in order to highlight the bands specific to chemical modification (Figure 2). This region is also specific to bands of tyrosine amino acid, a constitutive amino acid of silk fibroin that is related to PNIPAM binding. Literature data show that grafting redox systems based on ammonium cerium nitrate (ACN) in acid medium can generate radicals on carbons bound with hydroxyl groups [12,15]. The silk fibroin protein primary structure showed two potential amino acids with side hydroxyl groups liable for NIPAM monomer binding: serine and tyrosine. The tyrosine spectrum has gained more interest than serine amino acid, due to its ability to easily form radicals. The benzene ring of tyrosine can offer a higher stability for radicals due to its conjugation on multiple carbons. The assignment of tyrosine bands was made in correlation with the main literature attribution [31,32]. The main bands of SF-g-PNIPAM lie within the 756 cm^−1^ region and can be assigned to ring-breathing in the phase deformation of tyrosine ring. This band showed significantly increasing intensity with respect to the SF spectrum, due to the binding of NIPAM monomer directly with the tyrosine ring as it starts to vibrate more intensely. The next band at 1171 cm^−1^ can be assigned to in-plan C-H bond deformation with a contribution from benzene ring-C stretching. This band also showed an increasing intensity with respect to the SF spectrum due to the binding of NIPAM monomer. In can be deduced that this band assumed a direct binding of NIPAM monomer to the tyrosine ring via a C-C bond, due to the more intense vibration of the benzene ring-C stretching. The next band in the 1234 cm^−1^ region can be assigned to para-substituted benzene, with a totally symmetric stretching that reveals a significantly increasing intensity with respect to the SF spectrum. Furthermore, this band was related to the binding of NIPAM monomer in the orto-position aromatic ring. There can be bonding for both orto-positions, allowing the generation of symmetrical structures. The binding of NIPAM in the para position via a carbon bond with a hydroxyl group is in good agreement with the literature data, meaning that ACN redox system was able to generate radicals on such carbons [31,32,33]. The next band at the 1265 cm^−1^ region can be assigned to benzene ring-O stretching (20%) with a contribution from symmetric ring deformation (80%). Thus, there is a new band with respect to the SF spectrum, due to NIPAM grafting on the tyrosine benzene ring. All modified bands correlated with each other and confirmed the idea that NIPAM monomer binds on the tyrosine benzene ring in the orto-position aromatic ring through the generation of PNIPAM bridges. Considering the increasing intensity of the characterized bands, for a ratio of 0.1 *w*/*w* (NIPAM to SF), we can assume that the crosslinked silk fibroin network was obtained from PNIPAM bridges that generated strong vibrations in the tyrosine benzene ring.

**Figure 3:**
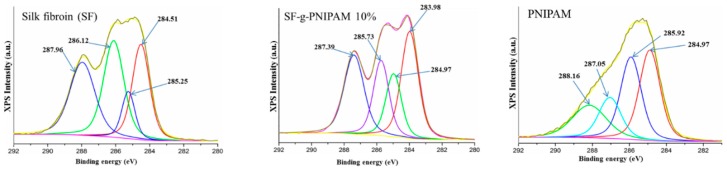
XPS spectra of silk fibroin (SF), SF-g-PNIPAM and PNIPAM.

XPS analysis data confirmed the grafting of NIPAM monomer onto silk fibroin, and XPS also supported the RAMAN results. The high-resolution spectra of C 1s are shown in Figure 3. XPS analysis was carried out for crude silk fibroin (SF) and SF-g-PNIPAM (ratio 0.1 *w*/*w* to SF). The C 1s-specific main peak revealed a split for both spectra. Both C 1s main peaks can be divided into four secondary peaks through deconvolution due to the complex carbon natures of protein structures. Thus, the silk fibroin spectra showed four secondary C 1s peaks: a first peak centered at about 284.51 eV, corresponding to C-C; a second peak centered at 285.25 eV, which can be assigned to C-H (CH_3_ in alanine; -CH_2_- and -CH- in backbone); a third peak centered at 286.12 eV, which can be assigned to C-O/C-N; and a fourth peak centered at 287.96 eV, which can be assigned to C=O/N-C=O [34]. The C 1s spectra of PNIPAM revealed four peaks: a first peak centered at about 284.97 eV, assigned to C-C contribution, and three deconvoluted peaks centered at 285.92, 287.05 and 288.16 eV, assigned to multiple C-H groups, C-O/C- and C=O/N-C=O, respectively [35,36]. The C 1s spectra of SF-g-PNIPAM (0.1 *w*/*w* to SF) also revealed four peaks shifted to lower values with respect to the silk fibroin spectrum: the first peak was centered at about 283.98 eV, which can be assigned to C-C, with the contribution of a new carbon species from PNIPAM structures [35,36]. Furthermore, the C-C peaks with lower values than 284.5–284 eV are usually associated with the sp^2^ atom range [37]. The sp^2^ hybridization was present in the benzene ring of amino acid tyrosine. These results confirm a new C-C species that is directly bonded to the benzene ring of tyrosine via the grafting of NIPAM monomer. The next deconvoluted peaks were centered at 284.97, 285.73 and 287.39 eV, and can be attributed to multiple C-H groups, C-O/C- and C=O/N-C=O, respectively, with a contribution from the PNIPAM structure due to a shift towards lower values. Furthermore, the results of the atomic percentage ratio for C-C and -CH- peaks in SF-g-PNIPAM, as compared to silk fibroin, were 1:1.2 and 1:1.7, respectively. These results represent the contribution of the new species from PNIPAM and the crosslinked network, which increased the number of new carbon bonds. The results are in good correlation with the RAMAN spectra results.

#### 2.1.1. DLS Analysis

The molecular weight (MW) of regenerated silk fibroin was obtained by detection of light scattering from interaction with protein molecules. In this study, the samples were exposed to a monochromatic wave of light using multiple detectors. Four diluted solution samples with different silk fibroin concentrations (0.05–0.1%) were tested. An average molecular weight of about 79,000 Da was determined.

#### 2.1.2. XRD Investigation

**Figure 4:**
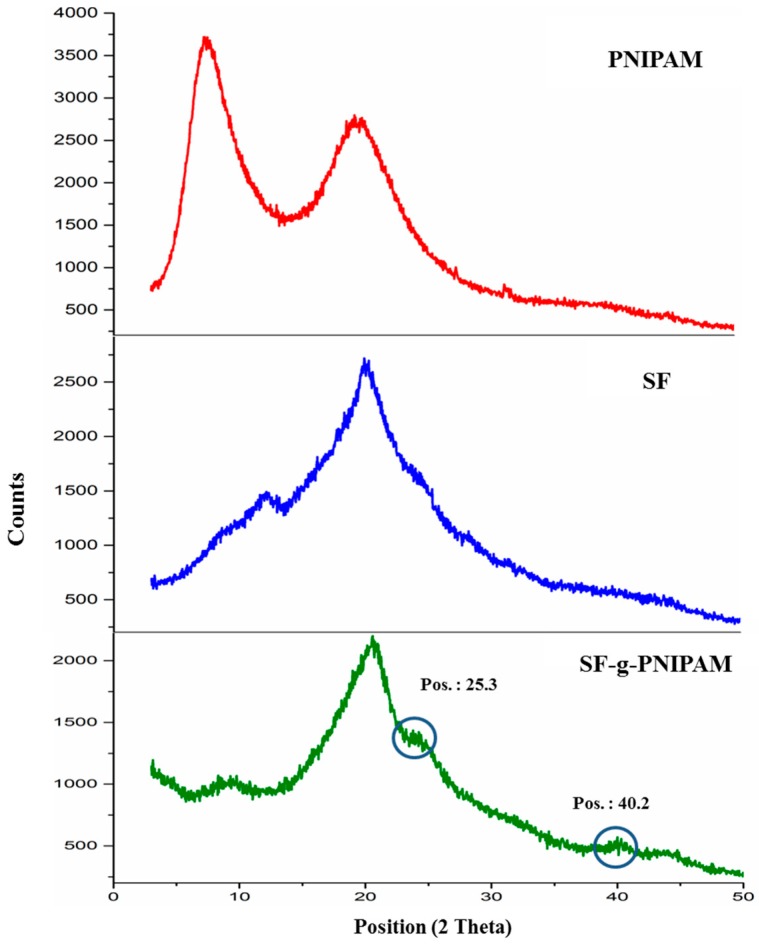
XRD diffractograms of silk fibroin (SF), PNIPAM and SF-g-PNIPAM.

XRD structural characterization of silk fibroin and modified samples corresponding to secondary structure is shown in Figure 4. The silk fibroin diffractogram revealed three main peaks at 2-theta angles of 12.21°, with a 20.29° characteristic for silk II (β-sheet) and a 24.21° characteristic for silk I [38,39]. The three peaks had a crystalline spacings of 7.24, 4.37 and 3.67 Å, respectively. The PNIPAM diffractogram showed two main peaks at 2-theta angles of 7.56°, with a 19.55° characteristic for crystallinity of the side amide bonds (which generate interactions to facilitate an ordered arrangement among polymer structures). The main peaks exhibited crystalline spacings of 11.68 and 4.53 Å, respectively. The modified silk-g-PNIPAM revealed a modification of the peak at 2-theta angles of 12.21° towards lower values of about 8.1°, due the contribution of a peak at 7.24° specific to the PNIPAM crystalline structure. Furthermore, the diffractograms showed an increasing intensity of the peak at 2-theta angles of 25.3°, probably resulting from the same modification of silk I corresponding to the PNIPAM grafting reaction.

The new 2-theta peak at 40.2° could be explained by a new ordered structure induced by the interaction between silk fibroin and PNIPAM, which is not typical for native silk structures. The main peak at 20.21° suffered no modification as a result of the PNIPAM grafting, and the results suggest that, similar to β-sheet, the secondary structure suffered minimal modification. The modification induced by PNIPAM probably occurred at the less ordered areas (amorphous) of the chains, leading to a more organized structure without any real modification of the entire crystalline degree of the silk protein.

### 2.2. Thermal Behavior of the SF and SF-g-PNIPAM

#### 2.2.1. DSC Analysis

**Figure 5:**
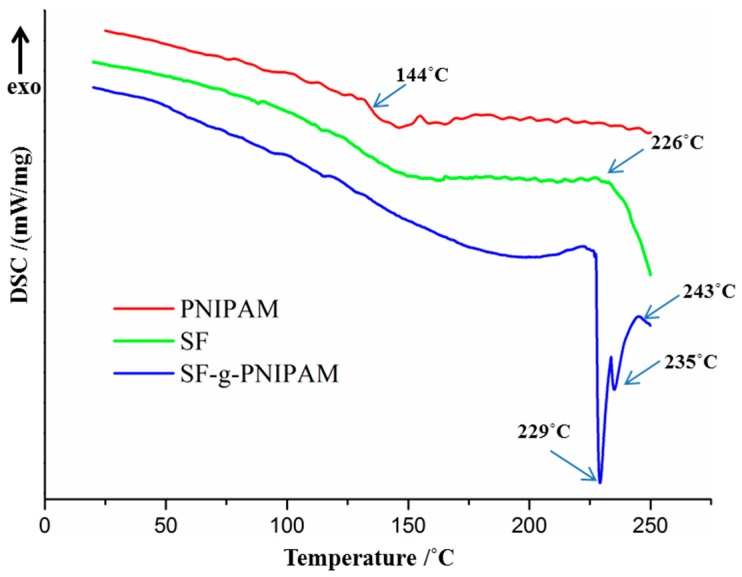
DSC curves of silk fibroin (SF), SF-g-PNIPAM and PNIPAM.

The thermal behavior of silk fibroin and SF-g-PNIPAM (ratio 0.1 *w*/*w* to SF) was evaluated by DSC investigation. Figure 5 shows the DSC curve for silk fibroin during the second run (the first run was carried out to remove the thermal memory and water traces), with a clear glass transition T_g_ in the region of 165 °C and an endothermic peak starting at 226 °C that was responsible for thermal degradation [40,41]. PNIPAM curve showed a glass transition temperature peak at 144 °C. Furthermore, the PNIPAM curve revealed no peak responsible for thermal degradation, as that would require a temperature above 300 °C. The DSC curve for SF-g-PNIPAM showed an almost negligible glass transition T_g_ at 195 °C, and an endothermic peak split at 229 °C and 235 °C (this is caused by conformational changes appeared in the crystalline phase of new structures, such as PNIPAM bridges preceding degradation) [42]. Thus, the conformational changes of PNIPAM bridges led to a delay of degradation for the native silk fibroin structure, and it seems that the degradation peak started at 243 °C. This behavior explains an apparent higher thermal stability of SF-g-PNIPAM with respect to SF, which occurred by increasing the temperature of the thermal degradation peak. The DSC analysis did not reveal any significant transition of the random coil to β structure (sheet/turn) by chemical crosslinking. The effects of the crosslinking on thermal behavior and characteristic temperatures such as glass transition (T_g_) are very complex. The literature shows different behaviors and influences of network structures on physical properties [43,44]. The effects most likely depend on every studied polymer without a certain temperature shifting towards a lower or higher value. The results of the DSC investigation showed an increase in glass transition temperature (T_g_), a new degradation peak and splitting of the thermal degradation at more than one stage [45,46].

#### 2.2.2. TGA Analysis

**Figure 6:**
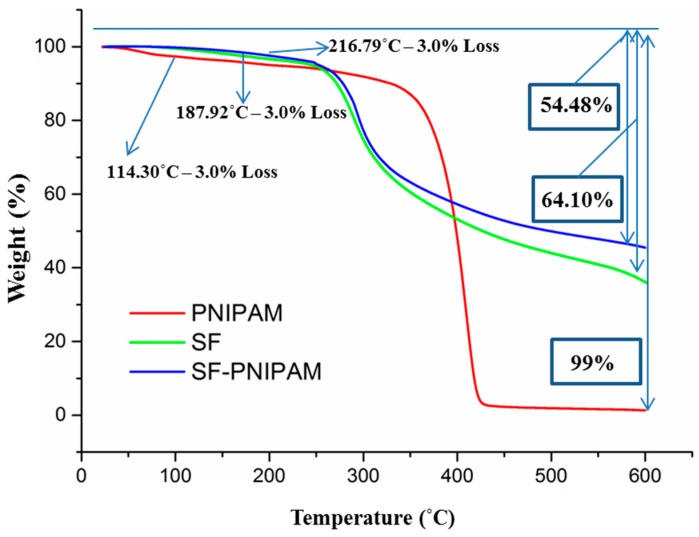
TGA curves of silk fibroin (SF), SF-g-PNIPAM and PNIPAM.

The thermal stability of the modified silk fibroin was directly related to the binding mode of the grafted NIPAM monomer within the silk fibroin structure. The thermogravimetric analysis, which investigated the thermal stability of regenerated silk fibroin and modified SF-g-PNIPAM (ratio 0.1 *w*/*w* to SF), is presented in Figure 6. Both samples showed a similar behavior among three subsequent regions in terms of shifting of temperature values. The first region occurred between 60–70 °C and 120–130 °C, due to the loss of the water traces. The second region occurred between 120–130 °C and 270–280 °C, with a slow mass loss probably due to the loss of the most volatile low molecular weight species coming from degradation. In this region, a mass loss of 3% was reached that was related to the temperature at which a polymer can be considered thermally stable. The silk fibroin showed a thermally stable temperature of about 188 °C, and SF-g-PNIPAM demonstrated a thermally stable temperature around 217 °C. This was probably due to a larger amount of water traces in the silk fibroin as compared to the modified one. Furthermore, the PNIPAM bridges led to an increase of the thermally stable temperature. The third region occurred between 270–280 °C and 600 °C, with a higher mass loss due to fibroin degradation resulting in gases such as carbon monoxide, carbon dioxide, hydrogen and nitrogen oxide [47]. The first step of this region could be attributed to the disintegration of the macromolecular side chains, which was likely followed by the main chain degradation and rearrangement of the carbon residues. PNIPAM revealed a lower thermal stability temperature at around 114 °C, but in fact thermal degradation appeared above 300 °C. PNIPAM demonstrated different thermal behavior than SF. Mass loss is significantly higher for SF, because PNIPAM displays a far simple chemical structure and does not lead to the generation of radicals that can react to one another, thus hindering nearly the entire mass loss. The SF-g-PNIPAM also had a lower mass loss rate and lower degradation percentage (54%) compared to the silk fibroin (64%), which was due to the crosslinking via PNIPAM bridges. These results suggest that the crosslinked network led to a slow increase in thermal stability for the SF-g-PNIPAM versus the crude silk fibroin.

### 2.3. Conformational Analysis by Circular Dichroism (CD)

**Figure 7:**
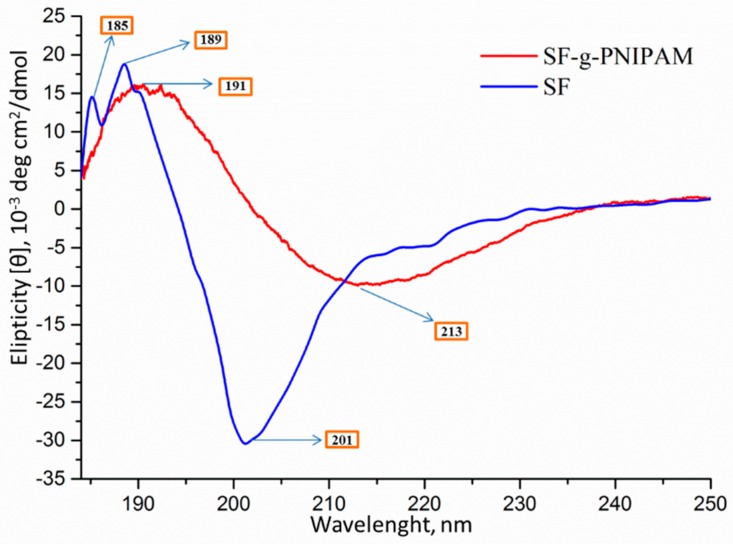
Circular dichroism (CD) spectra of silk fibroin (SF) and SF-g-PNIPAM.

The circular dichroism (CD) spectrum (Figure 7) showed a high negative peak at 201 nm, and two positive peaks at 185 and 189 nm, which are characteristic to protein secondary structure with a spatial arrangement by subunits of less-organized random coil and highly structured β-sheets [48]. These results were confirmed by quantitative estimation of the secondary structure. Thus, the quantitative estimation of the secondary structure was performed by integration calculation (secondary structure estimation (SSE)). The results highlight a random coil of 42.1%, highly organized β structures as β-sheet arrangement of 31.1%, β-turns of 14.6% and a right-hand α–helix of 12.2%. The spectrum of SF-g-PNIPAM revealed shifting of positive and negative peaks to higher values (191 and 213 nm, respectively). This behavior suggests that grafting with NIPAM may have led to a significant structural rearrangement by decreasing the percentage of β-sheets in favor of the α–helix percentage. The rearrangement was directly related to the influence of PNIPAM bridges, which probably interpose the highly ordered glycine-alanine-glycine-alanine-glycine-serine units of small size amino acids.

### 2.4. Solubility of Silk Fibroin-Grafted Poly-N-Isopropylacrylamide (SF-g-PNIPAM) and Characterization of the Crosslinked Network

**Figure 8:**
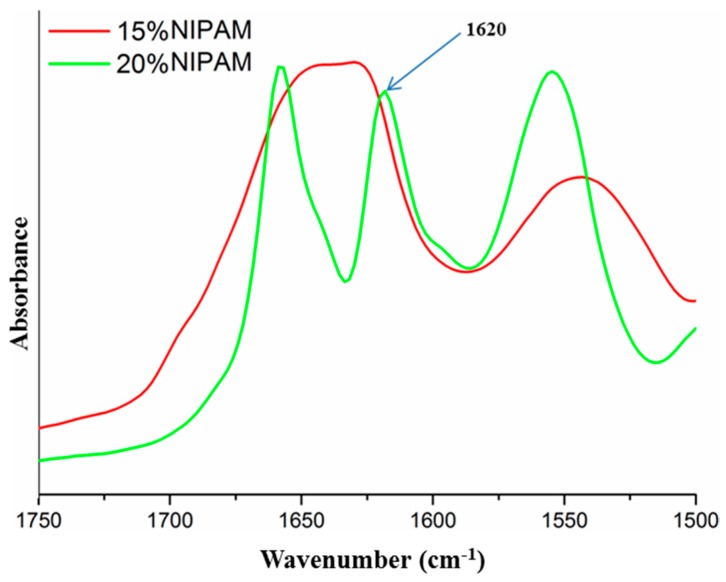
FTIR spectra of residual NIPAM monomer from different SF-g-PNIPAM samples).

The grafted silk protein showed no solubility in lithium bromide aqueous solution or other solvents for crude silk fibroin. The dissolution tests of SF-g-PNIPAM revealed that grafting generated a modification of the solving behavior due to crosslinking bridges. The first step in understanding the mechanism is to determine the maximum amount of NIPAM monomer that can be grafted onto the silk fibroin substrate. The samples were subjected to FTIR analysis to check the presence of residual monomer. The residual monomer could be seen in FTIR spectrum by the presence of a C=C peak at 1620 cm^−1^, which is characteristic only to monomer structure. The samples obtained by variation of NIPAM (5–30%) with constant ACN (5%) showed a residual monomer beginning at 10% NIPAM concentration. The samples obtained by variation of NIPAM (5–30%) and ACN (2.5–15%) showed a residual monomer starting at 15% NIPAM/7.5% ACN concentrations (Figure 8). This suggests that higher amounts of ACN initiator can influence the crosslinking degree, likely due to the generation of additional reaction sites (although with an upper limitation). These results are sustained by the swelling tests, which showed a significant decrease of the swelling degree when increasing the crosslinking agent up to a 15% NIPAM concentration. An increase of NIPAM concentration over 15% induced no further changes, suggesting that this concentration could be the limitation of the monomer amount as a crosslinking agent (Figure 8, Table 1).

**Figure 9:**
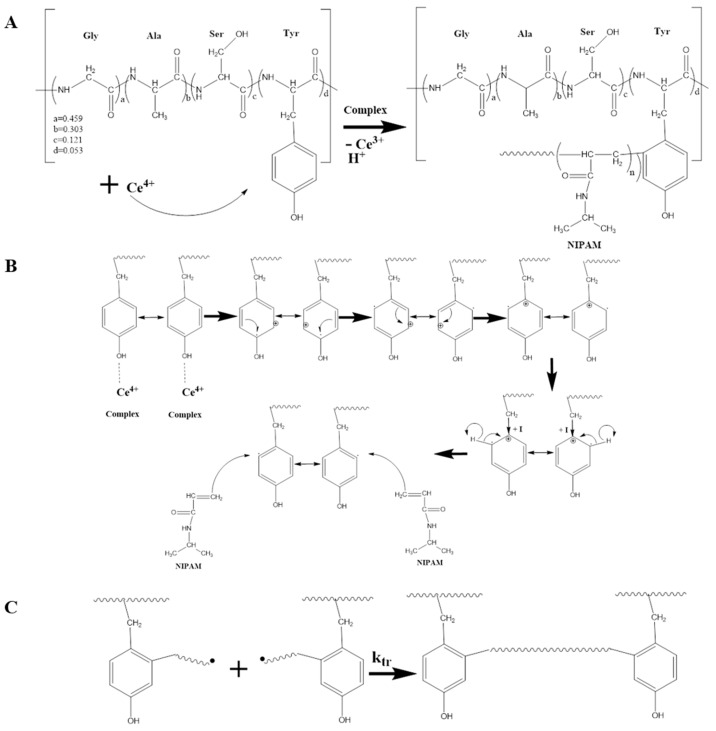
Mechanism of the initiation by cerium ammonium nitrate (**A**), propagation (**B**) and the termination reaction for PNIPAM (**C**).

The generation of reactive sites by the initiator (Figure 9) could even have been limited by the number of available reaction sites in the silk fibroin structure. By consulting the literature data regarding initiation sites generated by Ce^+4^ in the polymer chains, it was revealed that the most suitable point for radical generation is the aliphatic CH_2_-OH group, rather than –CH_2_-, due to the contribution of hydroxyl groups [12,13,15]. In the case of silk fibroin having amino acids with aromatic side chains such as tyrosine and phenylalanine, the stability of radical structures is higher for aromatics. The first amino acids are four times more present and may activate the aromatic structure through hydroxyl groups. Therefore, the proposed mechanism shows that tyrosine is the main starting point for PNIPAM growth (Figure 9A).

The detailed initiation and propagation mechanism require several exposures of stability alongside the aromatic ring of tyrosine amino acid. First, a critical condition is for cerium salt to be sustained in the form of Ce^4+^ and not reduced to Ce^3+^. During the solubilization process, Ce^4+^ is reduced to Ce^3+^ by interacting with water molecules that also generate hydrogen protons. The reaction is reversible under equilibrium and adding of strong acids such nitric acid will move the equilibrium to the left, sustaining the required Ce^4+^ concentration [12,49]. The mechanism involves the formation of a complex Ce^4+^ polymer. The complex leads to unimolecular disproportion via the generation of Ce^3+^, a free radical on substrate and a proton [49,50,51]. In the case of tyrosine substrate, an electron is most likely transferred to Ce^4+^ from a meta-position, generating a radical cation into the aromatic ring. This supposition is considered because the cation reaches the carbon bond with methyl group through an electromeric effect wherein stabilization occurs as the result of a positive inductive effect. In this case, the radical reaches the orto-position, a proton is released, the ring preserves the aromaticity and an active radical site is generated. The monomer head attacks the radical site and the initiation is complete (Figure 9B).

The mechanism of termination reaction for PNIPAM through free radical polymerization has been investigated in the literature [52,53]. The literature kinetics data regarding the PNIPAM termination mechanism take into account three possibilities, with different contributions depending on the reaction conditions. The literature concludes that it is difficult to quantify the exact contributions. If the recombination reaction is favored by a situation in which the macro-radicals are not collapsed by the high system viscosity or high concentration, it can be the main mechanism without excluding the other two. Furthermore, obtaining a crosslinked network SF-g-PNIPAM can be done generally through recombination. This fact suggests that termination via recombination makes a significant contribution (Figure 9C).

Starting from a silk fibroin molecular weight of about 79,000 Da, the number of tyrosine reactive sites in a chain composed of amino acids and the number of residual amino acids can be calculated [54]. An average molecular weight for a structural unit was calculated by averaging the percentage of amino acids and the molecular weight of each amino acid and reducing with amount of water eliminated by condensation:

(1) $$\left[u\right]=\overset{i=18}{\underset{i=1}{\sum }}\left(\%a_{i}*wa_{i}\right)-\frac{ww}{2}$$

where [*u*] is the average molecular weight of a structural unit of the chains, *%a* is the percentage of amino acids, *wa* is the molecular weight of the amino acid and *ww* is the molecular weight of water divided by two, since a molecule of water is eliminated by two amino acids. The average molecular weight of a structural chain unit had a calculated value of about 74.1 Da and it was correlated with the literature experimental values of about 74.4 Da [47]. This calculation allowed us to determine the average number of residual amino acids for a molecular weight of 79 kDa.

**Figure 10:**
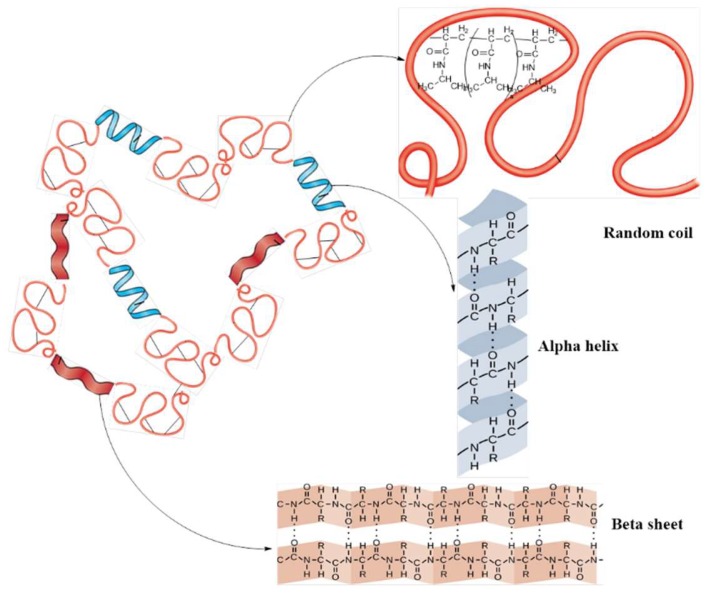
SF-g-PNIPAM crosslinked structure.

(2) MMW=[u]∗na

where *na* is the average number of amino acids present on silk fibroin chains. The calculated average number of amino acids was about 1066 for every chain. This result is in good correlation with the literature data with respect to average molecular weight (MW) [54]. Considering the tyrosine percentage [13], there was an average number of 56 tyrosine amino acids for every 1066 amino acids on every chain. The network characterization of polymers in a solvent solution is difficult to understand, and there are few tools to determine interactions and structure. The most manageable information that can be obtained is related to the average density of crosslinks. For a better characterization and understanding of the crosslinked network, a mathematical model developed by P.J. Flory was applied [55]. The mathematical model allowed the calculation of number-average molecular weights of the polymers between crosslinking sites:

(3) $$-\left[\ln\left(1-V_{2}\right)+V_{2}+\chi *V_{2}^{2}\right]=\left(\frac{V}{v*M_{c}}\right)*\left(1-\frac{2M_{c}}{M}\right)*\left(V_{2}^{1/3}-\frac{V_{2}}{2}\right)$$

where *V*_2_ is the volume fraction of polymer in a swollen polymer at equilibrium with a pure solvent, *V*_1_ is the molar volume of the solvent, *v* is the specific volume of the polymer (reciprocal of its density), *M* is the molecular weight of the polymer before crosslinking, and *M_c_* is the number-average molecular weight of polymer between crosslinked junctions. *χ* is a parameter that characterizes the interaction between the solvent and the polymer [55]. The average molecular weight of polymer between crosslinking sites, *M_c_*, was estimated to be 1700 Da. Considering the average number of tyrosine sites on silk fibroin chains, this estimation can have a realistic value only if these reactive sites are distributed on some domains only, such as semi-crystalline random coils. Otherwise. multiplying *M_c_* with tyrosine will result in a higher molecular weight. The model results can reveal only an estimated distance between random coil domains where bridges form (Figure 10). The highly organized secondary β-sheet structure was predominant in silk fibroin, as we have already presented in the circular dichroism (CD) section and supported with the literature data [54]. The organized structure contained predominantly repetitive glycine-alanine-glycine-alanine-glycine-serine units of small size amino acids. This ordered structure is also accompanied by less organized random coil interposition. This aspect suggests that larger amino acids such as tyrosine are present predominantly into semi-crystalline regions in silk structures [28,56].

### 2.5. SEM Analysis

**Figure 11:**
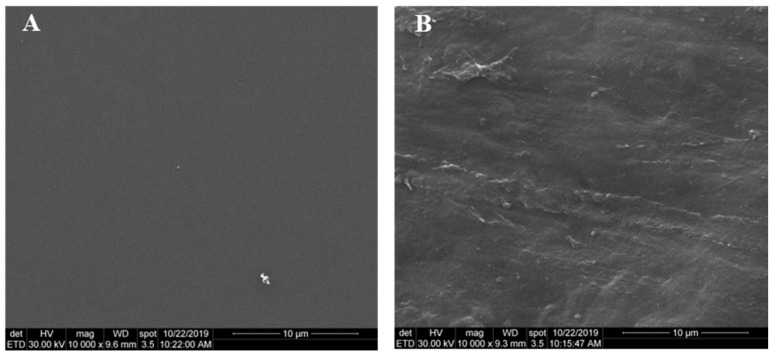
SEM images of crude silk fibroin ((**A**), SEI, ×10,000) and SF-g-PNIPAM ((**B**), SEI, ×10,000).

Morphological investigation of crude silk fibroin film and SF-g-PNIPAM is shown in Figure 11. Microphotographs depict secondary electron images (SEI) with 10,000× magnification. In Figure 11A, there is no difference in contrast in the image, which shows that the sample had a single phase with a uniform microstructure, which is characteristic to crude silk fibroin. In Figure 11B there are lines crossing the image from left to right that are not microstructural characteristics, as they represent the imprint of the support on which the sample was processed. This shows that PNIPAM induces a change in the microstructural arrangement of silk fibroin, which is in good agreement with the CD analysis and XRD investigation.

## 3. Materials and Methods

### 3.1. Materials

*Bombyx mori* silkworm cocoons were supplied by S.C Sericarom S.A. (Bucharest, Romania) as a source for the natural silk fibroin protein. Ammonium cerium nitrate (ACN), lithium bromide, sodium carbonate, sodium bicarbonate, sodium dodecyl sulfate, hydrochloric acid, sulfuric acid, nitric acid and *N*-isopropylacrylamide (NIPAM) monomer were provided by Sigma-Aldrich (St. Louis, MO, USA).

### 3.2. Methods

#### 3.2.1. Obtaining of Silk Fibroin Solution

Silk fibroin solution was obtained according to the following two steps: (i) silk fibroin was degummed to purified fibroin fiber; and (ii) the silk fibroin solution was prepared by dissolving the silk fiber. The degumming process consists of the removal of sericin and impurities to yield silk fibroin. Cocoons are first cut into pieces, then they are boiled in a mixture of water and salts as sodium carbonate, sodium bicarbonate and sodium dodecyl sulfate (as surfactant). The washing cycle is repeated several times until clean fibers are obtained. The purified fibers are washed in a high volume of distilled water in order to remove the surfactant and other salts, then the fibers are dried at 40 °C for 24 h. The silk fibroin solution was prepared fiber dissolution in a 9.5 M aqueous lithium bromide solution at 60 °C for several hours. The obtained silk fibroin solution was dialyzed against distilled water for one week to remove lithium and bromide ions. Stock solution was kept in the fridge before use.

#### 3.2.2. Synthesis of Silk Fibroin-Grafted Poly(*N*-Isopropylacrylamide) (SF-g-PNIPAM)

**Figure 12:**
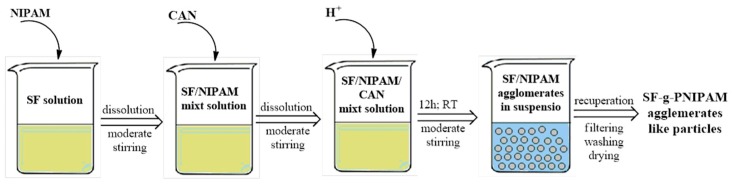
Scheme for obtaining SF-g-PNIPAM.

The grafting reaction of NIPAM monomer onto silk fibroin protein was carried out via radical polymerization mechanism based on redox reaction. The redox system was generated in the presence of ammonium cerium nitrate Ce^4+^ and an acid as oxidizing agent in the equilibrium (Ce^4+^/Ce^3+)^. Several ratios of acid/SF (*v*/*w*) were proposed including 0.1:11 for nitric acid; 5.5:21 for hydrochloric acid; and 1:28 for sulfuric acid. All the acid solutions had a concentration of 0.1 M. The reactions with hydrochloric acid and sulfuric acid showed no modification at lower ratios or degradation of SF at higher ratios. The reaction with nitric acid showed a grafting reaction at a lower ratio and protein degradation at a higher ratio. Briefly, NIPAM monomer (ratio 0.05 *w*/*w* to SF) and ammonium cerium nitrate (ACN) (ratio 0.025 *w*/*w* to SF and 0.5 *w*/*w* to NIPAM) were added to the silk fibroin solution. After total dissolution of the reactants into the SF solution, 0.1 M nitric acid was added (ratio 2.5 *v*/*w* to SF). The next synthesis involved increasing the amount of NIPAM monomer in order to reveal the modification of the grafting reaction (Table 1). Solutions were obtained with various NIPAM percentages from 0.1–0.3 *w*/*w* to SF, keeping the same acid nitric ratio for each (2.5 *v*/*w* to SF). Furthermore, solutions were obtained by increasing the concentration of ACN from 0.025–0.15 *w*/*w* to SF. The last synthesis modifications were carried out in order to show the ability of ACN to initiate the reaction mechanism (Table 2). The synthesis step began by adding NIPAM monomer to a fixed concentration of silk fibroin. After dissolution, the ACN reagent was added step by step to avoid protein precipitation. After several minutes of moderate stirring, nitric acid was added, and the reaction started. There was no required temperature for the reaction progress. The reaction lasted around 12 h and by the end agglomerations appeared in the media. These agglomerations appeared due to the lack of product solubility, as a result of the crosslinking process. The product was recovered by filtration and washing several times with distilled water. The product was dried overnight at 60 °C and an insoluble hydrogel powder was obtained. A synthetic scheme of the final product is shown below (Figure 12).

#### 3.2.3. Characterization Methods

##### FTIR Analysis

FTIR spectra of silk fibroin and grafted products were recorded on a Bruker Vertex 70 FT-IR spectrophotometer with an attenuated total reflectance (ATR) accessory with 32 scans and a 4-cm**^−^**^1^ resolution in the mid-IR region.

##### Raman Analysis

Raman spectra were recorded on a DXR Raman microscope from Thermo Fisher Scientific. The excitation laser wavelength was 532 nm using a laser power of 14 mW. The Raman spectra were collected in the range of 100–3200 cm^−1^, with a relevant display in the range of 500–2250 cm^−1^.

##### XPS Analysis

XPS spectra were recorded on a K-Alpha instrument from Thermo Scientific using a monochromated Al Kα source (1486.6 eV), at a pressure of 2 × 10^−9^ mbar. The deconvolution of C 1s was done to investigate the carbon type reaction.

##### Dynamic Light Scattering (DLS)

The average molecular weight of regenerated silk fibroin was determined by DLS in a static domain using a Malvern Zetasizer Nano instrument in the molecular weight module. The experiment was carried out using glass cuvettes with square apertures and toluene as the reference solvent. Water was used as the solvent for silk fibroin. Silk fibroin aqueous solutions were used for DLS analysis.

##### X-ray Diffraction (XRD) Analysis

XRD spectra were registered on a Panalytical X’PERT MPD X-ray Diffractometer in the range of 2θ = 10–80. An X-ray beam characteristic to Cu Kα radiation was used (λ = 1.5418 Å).

##### Differential Scanning Calorimetry (DSC)

A NETZECH Differential Scanning Calorimeter 204 was used to investigate the thermal and structural behavior of the samples. The analysis was performed in nitrogen atmosphere (99.99% purity) with a 10 °C/min heating rate and two complete heating–cooling cycles. Samples of 20 mg were weighed into aluminum pans, covered and fixed on the sample platform.

##### TGA Analysis

Thermogravimetric analysis was performed at a 10 °C/min heating rate in nitrogen atmosphere, from ambient temperature up to 600 °C, using TGA Q500 equipment (TA Instruments). The thermostability of the samples was evaluated at 3% mass loss.

##### Conformational Analysis by Circular Dichroism (CD)

Secondary structure of silk fibroin and grafted samples were evaluated using a Jasco J-1500 spectrophotometer, Japan (J-1500 Circular Dichroism Spectrophotometer), which has a quartz cell with a 1-mm path length. Every sample was scanned three times at 0.1% concentration in the range of 195–250 nm, with a scan rate 100 nm/min. Aqueous SF solutions and grafted specimen suspensions were used for CD analysis.

#### 3.2.4. Solubility and Crosslinking Effect of Silk Fibroin-Grafted Poly(*N*-Isopropylacrylamide) (SF-g-PNIPAM)

The solubility of the new grafted protein SF-g-PNIPAM was evaluated by dissolution into various solvents for silk fibroin, such as 10 M lithium bromide aqueous solution at 60 °C. The evaluation of the solubility was used to investigate the behavior modification induced by the grafting reaction to silk fibroin.

#### 3.2.5. SEM Analysis

The microstructure of the samples was analyzed by scanning electron microscopy (SEM) using a Quanta Inspect F50 containing a field emission gun (FEG) with a 1.2-nm resolution and an Energy Dispersive X-ray Spectrometer (EDS) with a 133-eV resolution at MnKα.

## 4. Conclusions

In this paper, the synthesis of a modified native silk fibroin and a synthetic polymer poly(*N*-isopropylacrylamide) was performed through a classical grafting reaction, but with the intention to obtain a crosslinked protein structure. The physico-chemical characterization supported a new way of bonding by using the reaction of an aromatic site from hydroxyl tyrosine instead of typical hydroxyl serine. Thus, the present research offers an interesting overview of the structuring of SF-g-PNIPAM based on the modification of chemical structure, spatial arrangement and crosslinking. The modified silk fibroin will be further tested for smart drug delivery systems. Certainly, this type of silk fibroin has the potential to play an important role in the development of nanoparticles loaded with hydrophilic drugs for controlled release applications.

## Figures and Tables

**Table 1:** Swelling degree of SF-g-PNIPAM samples with different monomer-to-initiator ratios.

Sample	Swelling Degree (%)
SF-g-PNIPAM (5% NIPAM/2.5% ACN)	360
SF-g-PNIPAM (10% NIPAM/5% ACN)	290
SF-g-PNIPAM (15% NIPAM/7.5% ACN)	200
SF-g-PNIPAM (20% NIPAM/10% ACN)	187
SF-g-PNIPAM (25% NIPAM/12.5% ACN)	181
SF-g-PNIPAM (30% NIPAM/15% ACN)	187

**Table 2:** Composition of the samples.

No.	Composition with Various Amount of NIPAM	Composition with Various Amount of NIPAM and Cerium Ammonium Nitrate (ACN)
1	SF-g-PNIPAM (5% NIPAM/5% ACN)	SF-g-PNIPAM (5% NIPAM/2.5% ACN)
2	SF-g-PNIPAM (10% NIPAM/5% ACN)	SF-g-PNIPAM (10% NIPAM/5% ACN)
3	SF-g-PNIPAM (15% NIPAM/5% ACN)	SF-g-PNIPAM (15% NIPAM/7.5% ACN)
4	SF-g-PNIPAM (20% NIPAM/5% ACN)	SF-g-PNIPAM (20% NIPAM/10% ACN)
5	SF-g-PNIPAM (25% NIPAM/5%ACN)	SF-g-PNIPAM (25% NIPAM/12.5%ACN)
6	SF-g-PNIPAM (30% NIPAM/5%ACN)	SF-g-PNIPAM (30% NIPAM/15%ACN)
